# Fibrinolytic Proteins and Factor XIII as Predictors of Thrombotic and Hemorrhagic Complications in Hospitalized COVID-19 Patients

**DOI:** 10.3389/fcvm.2022.896362

**Published:** 2022-06-10

**Authors:** Marina Marchetti, Patricia Gomez-Rosas, Laura Russo, Sara Gamba, Eleonora Sanga, Cristina Verzeroli, Chiara Ambaglio, Francesca Schieppati, Francesco Restuccia, Ezio Bonanomi, Marco Rizzi, Stefano Fagiuoli, Andrea D’Alessio, Grigorios T. Gerotziafas, Luca Lorini, Anna Falanga

**Affiliations:** ^1^Department of Immunohematology and Transfusion Medicine, Hospital Papa Giovanni XXIII, Bergamo, Italy; ^2^Hematology Service, Hospital General Regional Tecamac Instituto Mexicano del Seguro Social (IMSS), Mexico, Mexico; ^3^Department of Anesthesiology and Critical Care Medicine, Hospital Papa Giovanni XXIII, Bergamo, Italy; ^4^Unit of Infectious Diseases, Hospital Papa Giovanni XXIII, Bergamo, Italy; ^5^Department of Internal Medicine, Hospital Papa Giovanni XXIII, Bergamo, Italy; ^6^Medical Oncology and Internal Medicine, Policlinico San Marco – Gruppo San Donato, Bergamo, Italy; ^7^Sorbonne Université, INSERM UMR_S938, Research Group “Cancer-Hemostasis-Angiogenesis”, Centre de Recherche Saint-Antoine, Institut Universitaire de Cancérologie, Paris, France; ^8^School of Medicine and Surgery, University of Milano Bicocca, Milan, Italy

**Keywords:** COVID-19, thrombosis, bleeding, hypercoagulability, thromboelastometry, fibrinolysis, heparin, FXIII

## Abstract

**Introduction:**

In a prospective cohort of hospitalized COVID-19 patients, an extensive characterization of hemostatic alterations by both global and specific assays was performed to clarify mechanisms underlying the coagulopathy and identify predictive factors for thrombotic and hemorrhagic events during hospitalization.

**Materials and Methods:**

Intensive care unit (ICU; *n* = 46) and non-ICU (*n* = 55) patients were enrolled, and the occurrence of thrombotic and hemorrhagic events was prospectively monitored. At study inclusion, thromboelastometry together with the measurement of specific coagulation proteins and hypercoagulation markers was performed.

**Results:**

Patients (median age 67 years) showed significantly shorter clot formation time together with greater maximum clot firmness by thromboelastometry, increased levels of F1 + 2 and D-dimer, as biomarkers of hypercoagulability, and of procoagulant factors V, VIII, IX, XI, and fibrinogen, while FXIII was significantly reduced. The concentration of fibrinolytic proteins, tissue plasminogen activator (t-PA) and plasminogen activator inhibitor type 1 (PAI-1) were elevated in the overall cohort of patients. Many of these hemostatic alterations were significantly greater in ICU compared to non-ICU subjects and, furthermore, they were associated with inflammatory biomarker elevation [i.e., interleukin 6 (IL-6), C-reactive protein (CRP), neutrophil to lymphocyte ratio (NLR), and procalcitonin]. After enrollment, 7 thrombosis and 14 major bleedings occurred. Analysis of clinical and biological data identified increased t-PA, PAI-1, and NLR values as independent predictive factors for thrombosis, while lower FXIII levels were associated with bleeding.

**Conclusion:**

This study demonstrates alterations in all different hemostatic compartments analyzed, particularly in severe COVID-19 conditions, that strongly correlated with the inflammatory status. A potential role of fibrinolytic proteins together with NLR and of FXIII as predictors of thrombotic and hemorrhagic complications, respectively, is highlighted.

## Introduction

The clinical course of severe COVID-19 of hospitalized patients can be complicated by the occurrence of thromboembolic events, involving both venous and arterial districts. High incidence of pulmonary embolism (PE) (20–30% of cases), deep vein thrombosis (DVT), catheter-related thrombosis as well as arterial thrombosis such as ischemic strokes is reported (1–5). Thrombotic complications have been observed at higher rate in patients admitted to the intensive care units (ICUs) as compared to patients hospitalized in ward units (non-ICU). The causes of the increased rate include acute infectious and inflammatory reactions, together with prolonged immobility and hospital stay (6, 7), and the onset of a systemic hypercoagulable state.

Inflammation-driven systemic blood clotting activation is indeed present in SARS-CoV-2 patients, a mechanism that is mediated by the activity of inflammatory cytokines on the different compartments of the hemostatic system (8–10), including platelets (11, 12) and vascular endothelium. According to literature reports, COVID-19-associated hypercoagulable state is characterized by elevated levels of D-dimer, fibrinogen, factor VIII (FVIII), and von Willebrand factor (vWF), together with reduced ADAMTS-13 activity (13–15). Furthermore, vWF has been identified as a significant predictor of in-hospital mortality by SARS-CoV-2 infection (16).

In addition, also an increase of bleeding tendency has been described in COVID-19 patients (17, 18) that has been associated with the occurrence of thrombocytopenia (19, 20), coagulation factors consumption (21), and hyperfibrinolysis (22), all typical signs of disseminated intravascular coagulation (DIC)-like syndrome (23–25).

Given the morbidity and mortality impact of the thrombotic burden in COVID-19, the prevention of thrombotic complications is of fundamental importance. Recommendations and guidelines by national and international scientific societies all agree in the use of thromboprophylaxis with low molecular weight heparin (LMWH) or unfractionated heparin (UFH) in most patients (1, 26–31). However, occurrence of thrombotic complications was observed despite anticoagulant prophylaxis, leading to the choice of using intermediate- or full- (therapeutic) heparin doses to prevent thrombosis (27, 28, 32, 33). Notably, increasing anticoagulant doses might expose patients to an increased hemorrhagic risk (28, 29). Therefore, the identification of risk predictors of thrombosis and bleeding under anticoagulant treatment is essential for the selection of patients who may benefit from higher doses of anticoagulant prophylaxis. Until now, among biochemical parameters, D-dimer has been identified as a predictive factor for thrombosis in patients affected by this disease (6, 34, 35).

In this study, in a prospective cohort of consecutive patients hospitalized in both ICU and medical wards (non-ICU) for COVID-19 in Bergamo (Italy), we performed an extensive characterization of the hemostatic alterations by both global and specific assays, to: (1) clarify mechanisms underlying the coagulopathy in patients with different severity of the disease, (2) assess the occurrence of thrombosis and bleeding during hospitalization, and (3) identify clinical and laboratory biomarkers potentially predictive of these complications to be used in future clinical studies.

## Materials and Methods

### Study Subjects and Patient Classification

This two-center prospective study included 101 consecutive adult patients (≥18 years old) with a confirmed diagnosis of COVID-19 by RT-PCR on nasopharyngeal swabs and hospitalized in both ICU and non-ICU units. The patients were enrolled between 23rd March and 1st May 2020 at two Italian hospitals of the Bergamo area: Hospital Papa Giovanni XXIII and Policlinico San Marco/Gruppo San Donato. Patients were enrolled within 2 weeks from hospitalization. The study protocol was approved by the local Ethics Committee of Papa Giovanni XXIII Hospital that waived the need of informed consent and was conducted according to the last revision of the Helsinki Declaration. This study is part of the EMOCOVID study (registered at clinicaltrials.gov identifier # 04595110).

Clinical characteristics, age, gender, relevant comorbidities, body mass index (BMI), thrombotic and bleeding events, and the ratio of partial pressure of arterial oxygen and fractional concentration of oxygen inspired air (PaO2/FiO2) (36), were recorded. Anti-COVID-19 treatments included: antivirals, steroids, hydroxychloroquine, and tocilizumab. Anticoagulant treatment [i.e., heparins, vitamin K antagonists (VKAs), or direct oral anticoagulants (DOACs)] was provided based on the risk stratification of patients and guideline recommendations of anticoagulation in SARS-CoV-2 infection (37). During hospitalization, the occurrence of objectively confirmed thrombosis [i.e., DVT, pulmonary embolism (PE), arterial thromboembolism (ATE)], and bleeding were recorded until hospital discharge or in-hospital death.

Blood samples from a group of 108 (75M/33F) hospital employees with a median age of 49 years (range: 35–64 years) served as controls for coagulation testing, providing a specific informed consent for the use of their blood samples. Subjects were free of cardiovascular disease, thrombotic or bleeding disorders, diabetes, cancer, or infectious diseases, and were not taking anti-platelet, or anti-inflammatory drugs in the last 10 days before blood sampling, nor anti-coagulant drugs.

### Blood Samples

Peripheral venous blood was drawn using a 21-ga needle. After discarding the first 2–3 mL, blood was collected into evacuated tubes with no additive (BD Vacutainer^®^ SST*™* II, Becton, Dickinson) for C-reactive protein (CRP) and procalcitonin, in tubes containing trisodium citrate (0.129 M, 1:9 vol:vol) for coagulation studies (BD Vacutainer^®^ Blood Collection Tubes, Becton, Dickinson), and in tubes containing K3-ethylenediamine tetraacetic acid (K3-EDTA, 7.2 mg BD Vacutainer) for the complete blood cell count study. Within 2 h from blood collection, citrated plasma was isolated by a 2-step centrifugation at 3000 × *g* for 15 min at 25°C, divided in aliquots and stored at −80°C until testing at the Laboratory of Hemostasis and Thrombosis (Hospital Papa Giovanni XXIII, Bergamo, Italy). Blood sampling, plasma preparation, and storage have been conducted according to standardized procedure and international recommendations (38).

### Hematological and Inflammatory Parameters

White blood cell differential count, hematocrit, hemoglobin, red blood cell and platelet counts were determined by a Sysmex-XE 2100 hematology analyzer (Sysmex, Kobe, Japan). CRP and procalcitonin were measured by an immunoturbidimetric assay on ADVIA 2400 (Siemens Healthcare Diagnostics, United States), while interleukin 6 (IL-6) plasma levels were assayed by ELISA kit (BioSource/Invitrogen Hu) according to the manufacturer’s instructions.

### Thromboelastometry

Global hemostatic potential was measured by thromboelastometry (ROTEM^®^, Werfen, Italy) in citrated whole blood, according to the instructions provided by the manufacturer, using EXTEM reagent to activate the extrinsic pathway, and INTEM reagent to activate the intrinsic pathway. The impact of fibrinogen on clot firmness was tested using the platelet inactivating FIBTEM reagent. All samples were run for 60 min. Parameters considered were: clotting time (CT), clot formation time (CFT), maximum clot firmness (MCF, maximum tensile strength of the thrombus), and maximum lysis time (ML).

### Coagulation Assays

Prothrombin time (PT, STA-NeoPTimal), activated partial thromboplastin time (aPTT, STA-Cephascreen, STA-C.K. Prest), fibrinogen by Clauss method (STA-Liquid Fib), protein C activity (PC, STA-STAChrom Protein C), free protein S (FPS, STA-Liatest Free Protein S), and antithrombin activity (AT, STAChrom AT) were measured on the STA Compact Max 3 coagulation analyzer. Plasma levels of FII, FV, FVII, FIX, FX, FXI, FXII, FVIII, FXIIIa (HemosiIL FXIII Ag), and anti-FXa activity (HemosIL Liquid Anti-Xa) were assessed on the ACL TOP 500 coagulation analyzer (Werfen group, Italy). All assays were performed according to the manufacturer’s instructions.

### Markers of Hypercoagulation

Plasma levels of prothrombin fragment 1 + 2 was determined by a commercially available ELISA (F1 + 2, Enzygnost^®^, Siemens Healthcare Diagnostics), while D-dimer on an ACL TOP 500 coagulation analyzer with the HemosIL D-dimer HS reagent (Werfen group).

### Fibrinolytic Proteins

Plasminogen activator inhibitor type 1 (PAI-1 Antigen, Zymutest, Hyphen BioMed), and tissue plasminogen activator (t-PA, Zymutest, Hyphen BioMed) were determined in plasma samples by commercially available ELISA.

### Statistical Analysis

In the descriptive statistics, categorical variables were summarized as frequencies and proportions, while continuous variables as median and 5th–95th percentile range or mean and SD, according to their distribution. Normally and non−normally distributed quantitative data were then compared using the unpaired Student’s *t*−test and Mann–Whitney *U* test, respectively; the Pearson’s Chi-squared test was applied to test differences between sets of categorical variables. Significant association between two variables were tested by the Pearson’s correlation coefficient and linear regression analyses. Predictors of thrombotic and hemorrhagic events were analyzed by the univariable and multivariable Cox regression analysis. Backward variable selection was performed considering as possible predictors laboratory and clinical parameters. Survival functions were estimated using the Kaplan–Meier method assuming the day of study enrollment as baseline time, while survival analyses were performed using Cox’s proportional hazard (PH) model. A two-sided *p*-value of 0.05 or less was considered statistically significant. Statistical analysis was performed by using IBM SPSS Statistics, Version 26.0. (IBM Corp.) and the Prism software version 8 (GraphPad software, Inc.).

## Results

### Clinical Characteristics of the Study Cohort

One hundred and one patients with COVID-19 admitted to both ICU (46%) and non-ICU (55%) were consecutively enrolled into the study and prospectively followed-up for thrombo-hemorrhagic events. Demographics and clinical characteristics of patients at the time of study inclusion are shown in Table 1. Patients had a median age of 67 years (range: 35–89 years), and 73% were male. At study enrollment, median PaO2/FiO2 ratio values were 108 mmHg (IC 95%; 57–223 mmHg). Specific COVID-19 medications consist of antivirals (33% of patients), steroids (41%), hydroxychloroquine (44%), and tocilizumab (12%).

**TABLE 1:** Characteristics of the study cohort at enrollment.

	Total cohort (*n* = 101)	No ICU (*n* = 55)	ICU (*n* = 46)	*p*-value
**Male gender *n* (%)**	73 (73)	38 (69)	35 (76)	0.289
**Age [years (mean/SD)]**	67 ± 12	71 ± 12	62 ± 9	0.013
Comorbidities (%) Type 2 diabetes Arterial hypertension Cardiopathy (atrial fibrillation) Active cancer Other	14 (14) 42 (42) 10 (10) 11 (11) 4 (4)	12 (22) 23 (42) 4 (7) 7 (13) 1 (2)	2 (4) 19 (41) 6 (13) 4 (9) 3 (7)	0.025 0.514 0.508 0.742 0.352
**PaO2/FiO2 mmHg [median (CI: 95%)]**	108 (57–223)	134 (86–208)	103 (55–225)	0.179
COVID-19 treatment (%) Antivirals Steroids Hydroxychloroquine Tocilizumab	33 (33) 41 (41) 44 (44) 12 (12)	5 (9) 18 (33) 12 (22) 1 (2)	28 (61) 23 (50) 32 (70) 11 (24)	0.005 0.154 0.065 0.145
**Antithrombotic strategy *n* (%)** LMWH • Prophylactic dose • Therapeutic dose UFH • Prophylactic dose • Therapeutic dose Fondaparinux VKA DOAC Antiaggregant	60 (60) 18 (30) 42 (70) 10 (10) 6 (60) 4 (40) 2 (2) 8 (8) 6 (6) 31 (31)	30 (55) 13 (24) 17 (31) 2 (4) 3 (6) 0 (0) 0 (0) 5 (9) 2 (4) 14 (25)	30 (65) 5 (11) 25 (54) 8 (17) 3 (7) 4 (9) 2 (4) 3 (7) 4 (9) 17 (40)	0.082 0.485 0.195
**Hemochromocytometric analysis** Leukocytes (10^9^/L) (*n*: 4.2–9.4) Neutrophils (10^9^/L) (*n*: 2.0–6.7) Lymphocytes (10^9^/L) (*n*: 1.13–3.40) Hematocrit (%) (*n*: 37.9–46.1) Platelets (10^9^/L) (*n*: 140–400) Neutrophil-lymphocyte ratio (NLR) (*n*: 1.2–2.1)	9.0 (4.1–22) 6.7 (2.3–19.3) 1.0 (0.3–2.5) 33 ± 6 200 (69–520) 8 (1.7–24.4)	7.7 (3.3–21.4) 5.9 (1.9–18.6) 1.2 (0.4–2.6) 34 ± 6 179 (59–473) 5 (1.5–16.3)	10.6 (5.0–22.6) 8.7 (3.9–20.9) 0.8 (0.2–2.4) 32 ± 5 275 (80–549) 11.4 (4.1–36)	<0.001 <0.001 0.003 0.344 <0.001 <0.001
**Routine coagulation assays** PT ratio (normal: <1.2) aPTT ratio (normal: <1.2)	1.2 (1.0–3.3) 1.3 (0.9–2.1)	1.2 (1.0–3.4) 1.2 (0.9–1.7)	1.1 (1.0–2.5) 1.4 (0.9–2.4)	0.808 0.009

*Data are presented as number (percentage) and shown for the entire cohort and according to disease severity. For hematocrit, data are presented as mean and SD, while for the rest of variables data are presented as median and 5th and 95th percentiles. p-Value by Mann–Whitney U test between patients hospitalized in non-ICU vs. ICU. LMWH, low molecular weight heparin; UFH, unfractionated heparin; VKA, vitamin K antagonists; Antiaggregant, acetylsalicylic acid 100 mg/day or clopidogrel 75 mg/day; PT, prothrombin time; aPTT, thromboplastin activated time.*

At study inclusion, 86% of the patients were receiving some anticoagulant strategy, including LMWH in 60 subjects (18 at prophylactic and 42 at therapeutic doses), UFH in 10 subjects (6 at prophylactic and 4 at therapeutic doses), fondaparinux at a prophylactic dose in 2 subjects, VKA for specific indications in 8 subjects, and DOACs in 6 subjects (1 rivaroxaban, 1 edoxaban 2, apixaban, and 2 dabigatran). Moreover, 31 (31%) patients had an antiplatelet therapy, that was aspirin in most cases (Table 1). Twenty-nine patients were receiving anticoagulant therapy for a thrombosis that occurred at hospitalization (10 patients with PE) or during hospitalization (19 patients), all before enrollment in this study.

### Routine Hematological Parameters

Table 1 shows hematological parameters measured at study entry. Leukocyte count over the upper reference value was observed in 49% patients and was associated with neutrophilia in 48% and lymphopenia in 51% of them. Significantly (*p* < 0.001) higher total leukocyte count was present in ICU compared to non-ICU patient group. Twenty-seven (27%) patients (8 ICU and 19 non-ICU) had mild-moderate thrombocytopenia, with a median platelet count of 115 × 10^9^/L (IC 95%; 24–149 × 10^9^/L). PaO2/FiO2 values, measured on the same day of blood sampling, were inversely (*p* = 0.003) related to both total leukocyte (*r* = −0.473) and neutrophil (*r* = −0.488) counts.

Prothrombin time ratio was prolonged (i.e., >1.2) in 49 patients, especially in those on VKA (*r* = 0.709, *p* < 0.001), that showed a median PT ratio of 2.5 (95% IC: 1.46–4.5). Moreover, aPTT was prolonged (i.e., >1.2) in 57 patients and was associated with heparin administration. Additionally, most patients (72%) showed hyperfibrinogenemia (i.e., fibrinogen values >400 mg/dL).

**FIGURE 1:**
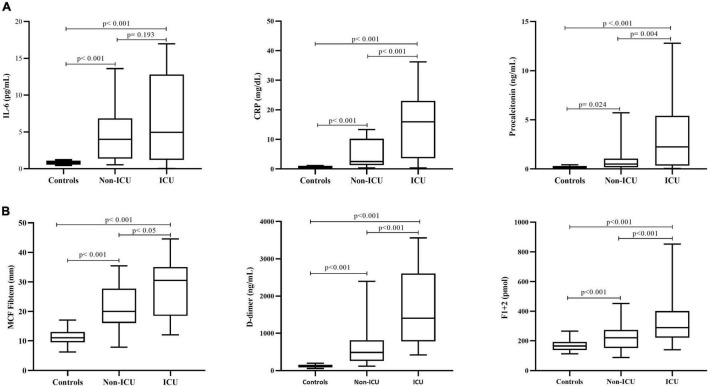
Biomarkers of inflammation and hypercoagulability. Circulating levels of inflammatory **(A)** and hypercoagulation **(B)** biomarkers in the overall cohort of COVID-19 patients according to disease severity (ICU vs. non-ICU) and compared to a group of healthy control subjects. IL-6, interleukin 6; CRP, C-reactive protein; MCF, maximum clot firmness. Data are expressed as median and 5th and 95th percentiles. *P* is statistical significance by Mann–Whitney *U* test, for the comparison of parameters among controls and hospitalized patients in non-ICU vs. ICU.

Finally, the analysis of inflammatory biomarkers, showed serum levels of IL-6 and C reactive protein (CRP) above the upper normal reference value in 25 and 78% of patients, respectively, while procalcitonin higher than 0.5 ng/mL was present in 62% of subjects. A significant and positive correlation was observed between CRP levels and fibrinogen (*r* = 0.566; *p* < 0.001) (Figure 1A). The neutrophil to lymphocyte ratio (NLR) was significantly augmented in the overall cohort of patients, with the highest values occurring in ICU subjects. Significant correlations (*p* < 0.001) were obtained between NLR and CRP (*r* = 0.403), procalcitonin (*r* = 0.512), and fibrinogen levels (*r* = 0.513).

### Global Hemostasis Evaluation by Thromboelastometry

Whole blood global hemostasis analysis by INTEM and EXTEM tests showed significantly (*p* < 0.001) shorter clotting formation time (CFT) and increased MCF values in patients compared to controls. Differently, CTs by EXTEM were significantly prolonged in patients compared to controls. Moreover, significantly increased MCF values were also obtained by FIBTEM test, the assay that specifically measures the contribution of fibrinogen and FXIII to the clot strength (Figure 1B). No lysis of the clot was observed in all the studied population, as demonstrated by normal lysis time (LT) values. In addition, ICU subjects displayed significantly higher MCF by FIBTEM compared to non-ICU subjects (*p* = 0.026), with values significantly associated with fibrinogen levels (*r* = 0.637, *p* < 0.001).

### Coagulation Profile Characterization

As shown in Table 2, compared to controls, the overall group of patients showed significantly (*p* < 0.001) higher levels of FV, FVIII, FIX, FXI together with significantly (*p* < 0.001) decreased levels of FII, FVII, FX, and FXIII. No differences were observed in FXII values. According to disease severity groups, ICU patients displayed significantly higher FV, FVIII, FX, and FIX and lower FXIII levels compared to non-ICU patients. Moreover, significant correlations were found between FVIII with CFT (*r* = −0.291; *p* = 0.040) by EXTEM, and with MCF by both EXTEM (*r* = 0.393; *p* = 0.005) and INTEM (*r* = 0.467; *p* = 0.001). Finally, PC and AT levels were significantly (*p* < 0.05) reduced while free-PS was slightly but significantly elevated as compared to control values, without differences according to disease severity.

**TABLE 2:** ROTEM parameters, plasma levels of coagulation factors/inhibitors, and fibrinolytic proteins overall and according to disease severity.

	Controls (*n* = 108)	Patients (*n* = 101)	*p*-Value	Non-ICU (*n* = 55)	ICU (*n* = 46)	*p*-value
**ROTEM analysis**						
CT EXTEM (s)	55 (44–70)	67 (50–122)	<0.001	64 (48–145)***	68 (50–119)***	0.379
CFT EXTEM (mm/s)	114 (83–151)	70 (42–149)	<0.001	84 (43–160)***	66 (40–143)***	0.055
MCF EXTEM (mm)	61 (54–68)	70 (53–80)	<0.001	68 (52–78)***	71 (56–82)***	0.068
CT INTEM (s)	160 (141–188)	167 (136–229)	0.046	164 (127–236)	175 (142–224)**	0.076
CFT INTEM (mm/s)	71 (52–103)	53 (38–115)	<0.001	54 (39–120)***	49 (35–101)***	0.214
MCF INTEM (mm)	63 (55–69)	72 (57–83)	<0.001	69 (57–80)***	73 (59–84)***	0.041
MCF FIBTEM (mm)	11 (5–17)	25 (9–42)	<0.001	21 (8–42)***	30 (9–42)***	0.889
**Procoagulant factors**						
FII (%)	99 ± 9	86 ± 27	<0.001	81 ± 27***	92 ± 25	0.031
FV (%)	94 ± 13	103 ± 31	0.009	91 ± 24	116 ± 33***	<0.001
FVII (%)	101 ± 12	84 ± 33	<0.001	76 ± 30***	94 ± 33	0.007
FX (%)	99 ± 7	83 ± 25	<0.001	77 ± 27***	91 ± 19**	0.008
FVIII (%)	102 (57–143)	194 (88–434)	<0.001	169 (88–310)***	234 (90–480)***	0.004
FIX (%)	105 ± 14	150 ± 61	<0.001	131 ± 52**	172 ± 64***	0.001
FXI (%)	97 ± 10	119 ± 42	<0.001	112 ± 40**	126 ± 43***	0.122
FXII (%)	97 ± 10	95 ± 34	0.605	92 ± 35	99 ± 32	0.270
FXIII (%)	110 (89–145)	59 (22–99)	<0.001	73 (33–111)***	40 (18–73)***	<0.001
**Anticoagulant proteins**						
PC (%)	117 (89–142)	103 (55–200)	0.008	100 (42–183)***	119 (57–200)	0.038
FPS (%)	95 (73–122)	107 (43–147)	0.007	102 (38–144)	115 (56–149)***	0.069
AT (%)	97 ± 14	96 ± 18	0.250	91 ± 21	94 ± 17	0.500
**Fibrinolytic proteins**						
t-PA (ng/mL)	5.3 (1.4–11)	20 (6–40)	<0.001	21 (9.0–43)***	18 (5.1–37)***	0.205
PAI-1 (ng/mL)	11 (2.7–32)	27 (10–64)	<0.001	29 (10–64)***	24 (6.0–77)***	0.269

*Data are presented as mean and SD or as median and 5th and 95th percentiles depending on their distribution. p-Value is statistical significance by Mann–Whitney U test to compare values between all cohort of COVID-19 patients vs. controls, and to compare values between non-ICU vs. ICU patients, after excluding patients with a previous thrombosis at enrollment.*

**p < 0.05, ***p < 0.001 vs. healthy control subjects.

### Hypercoagulation Biomarkers and Fibrinolytic Proteins

Compared to controls, increased levels of F1 + 2 and D-dimer were detected in both ICU and non-ICU patients (Figure 1B), with significantly higher values in ICU subjects, also after exclusion of those patients who experienced thrombosis before enrollment. A significant correlation was found between F1 + 2 and IL-6 (*r* = 0.701; *p* = 0.016) levels. As shown in Table 2, plasma levels of both t-PA and PAI-1 were significantly increased in patients compared to controls, without differences within ICU vs. non-ICU patients, with a significant correlation between the two proteins (*r* = 0.573; *p* < 0.001).

### Coagulation/Hematological/Biochemical Parameters According to COVID-19 Targeted Therapies

Table 1 provides a description of the different parameters evaluated in this study according to COVID-19 therapies that the patients were taking at the time of enrollment in the study. Patients on antiviral treatment (*n* = 33) displayed significantly (*p* = 0.017) higher platelet count [253 × 10^9^/L (IC 95%: 66–529)] compared to patients not on antiviral treatment [187 × 10^9^/L (IC 95%: 41–530)]. Differently, patients on hydroxychloroquine had higher levels of FXII (104 ± 30 vs. 79 ± 21%; *p* = 0.005), AT (97 ± 17 vs. 80 ± 21%; *p* = 0.015), and platelet counts [253 × 10^9^/L (IC 95%: 95–519) vs. 179 × 10^9^/L (IC 95%: 39–490)] compared to patients not receiving this treatment. Furthermore, hydroxychloroquine-treated subjects showed shorter aPTT ratio [1.31 (IC 95%: 0.9–2.1) vs. 1.7 (IC 95%: 0.9–3.2); *p* = 0.023] than patients without this drug, independently of gender, age, and heparin use (*B* = −0.350; *p* = 0.045). Regarding inflammatory parameters, patients on hydroxychloroquine treatment showed lower levels of IL-6 [44.7 pg/mL (IC 95%: 2–424) vs. 468 pg/mL (IC 95%: 332–603)] than patients not on this drug. Finally, tocilizumab-treated patients showed significantly (*p* < 0.05) lower levels of both CRP [5.2 mg/dL (IC 95%: 0.10–22) vs. 13.7 mg/dL (IC 95%: 0.3–37)] and procalcitonin [0.13 ng/mL (IC 95%: 0.04–0.92) vs. 0.40 ng/mL (IC 95%: 0.03–12)].

### Predictors of Thrombotic Complications During Follow-Up

After enrollment into the study, 7 thrombotic events (1 DVT, 5 EP, 1 ATE) occurred during follow-up, with a median time to event of 6 days (range: 3–12 days). All were first thrombosis. The measurement of heparinemia in plasma samples collected at study enrollment, showed levels of anti-FXa below the expected target in all the 7 patients that developed a thrombotic event [i.e., 1 patient on prophylactic LMWH = 0.10 IU/mL, 4 patients on therapeutic LMWH = 0.37 IU/mL (95% IC: 0.27–0.41), and 1 patient on therapeutic UFH = 0.31 IU/mL].

**FIGURE 2:**
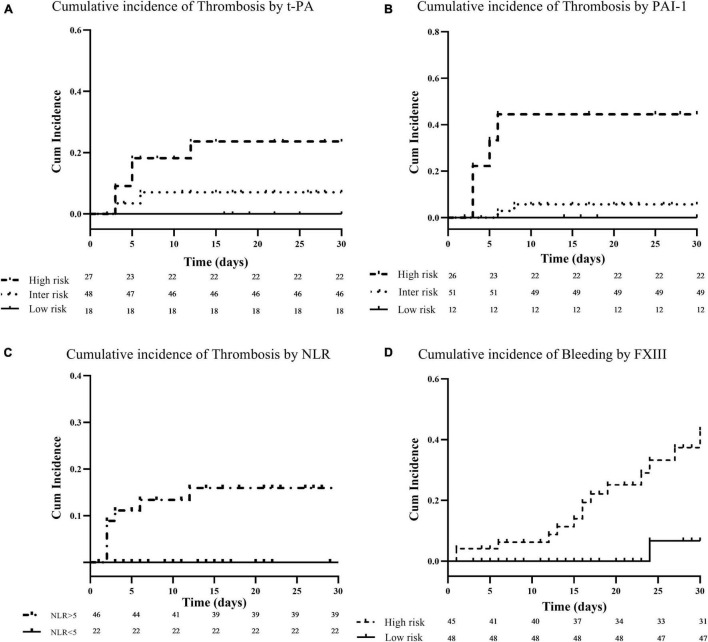
Cumulative incidence of thrombotic and bleeding complications. Panels **(A–C)** show Kaplan–Meier survival curves for thrombosis in relation to baseline plasma levels of t-PA, PAI-1, and neutrophil to lymphocyte ratio (NLR), respectively. Baseline t-PA and PAI-1 levels were categorized for visualization at the 25th and 75th percentile of their distribution in the patient cohort. Patients with biomarkers levels at or above the 75th percentile (high-risk) are compared to patients below the 25th percentile (low-risk), or within the 25th and 75th percentile (intermediate-risk). For NLR, patients were stratified according to a cut-off value of 5, and patients with an NLR ≥5 (high-risk) are compared to those with NLR <5 (low-risk). Finally, panel **(D)** shows Kaplan–Meier survival curve for bleeding events according to FXIII levels. Baseline FXIII levels were dichotomized at the 50th percentile of their distribution in the patient cohort (i.e., 60%). Patients with biomarkers levels at or above the 50th percentile (low-risk) are compared to patients below the 50th percentile (high-risk).

Among the different hemostatic and inflammatory parameters investigated in the present study, by Cox regression univariable analysis, we found that high levels of PAI-1 (HR: 1.043; CI: 95%: 1.007–1.080; *p* = 0.020), t-PA (HR: 1.091; CI: 95%: 1.016–1.171; *p* = 0.016), and NLR (HR: 1.089; CI: 95%: 1.032–1.149; *p* = 0.002) at enrollment were significantly associated with the development of thrombosis during follow-up. After stratification of patients according to the 25th (4 ng/mL) and 75th (32 ng/mL) percentiles of t-PA values distribution in the cohort of patients, three groups of subjects at different thrombotic risk were identified by Kaplan–Meier survival analysis (Figure 2A). Specifically, the cumulative incidence of thrombosis was 0% for the low-risk (t-PA ≤4 ng/mL), 8% (95% CI: 2.7–18%) for the intermediate (t-PA: >4 to <32 ng/mL), and 34% (95% CI: 6.9–62%) for the high-risk group (t-PA ≥32 ng/mL), with and HR of 4.7 (CI: 95%: 0.88–25.2; *p* = 0.057). By the same analysis, using the 25th (27 ng/mL) and 75th (42 ng/mL) percentiles of PAI-1 distribution values, the three groups of patients showed a thrombotic risk of 0% in the low-risk group (PAI-1 ≤27 ng/mL), 7.4% (95% CI: 2.6–17.4%) (PAI-1: >27 to <42 ng/mL) in the intermediate group, and 43% (95% CI: 10.8–75%) (PAI-1 ≥42 ng/mL) for the high-risk group, providing an HR of 7.5 (CI: 95%: 1.4–40.1; *p* = 0.021) (Figure 2B). Additionally, according to the pathological value of NLR (>5) (39), two groups of patients at different risk of thrombosis were identified, with a cumulative incidence of thrombosis of 0 and 23% (95% CI: 6.5–32%) for normal (<5) and pathological (≥5) of NLR values, respectively (HR: 1.098, CI: 95%: 1.042–1.157; *p* < 0.001) (Figure 2C).

**FIGURE 3:**
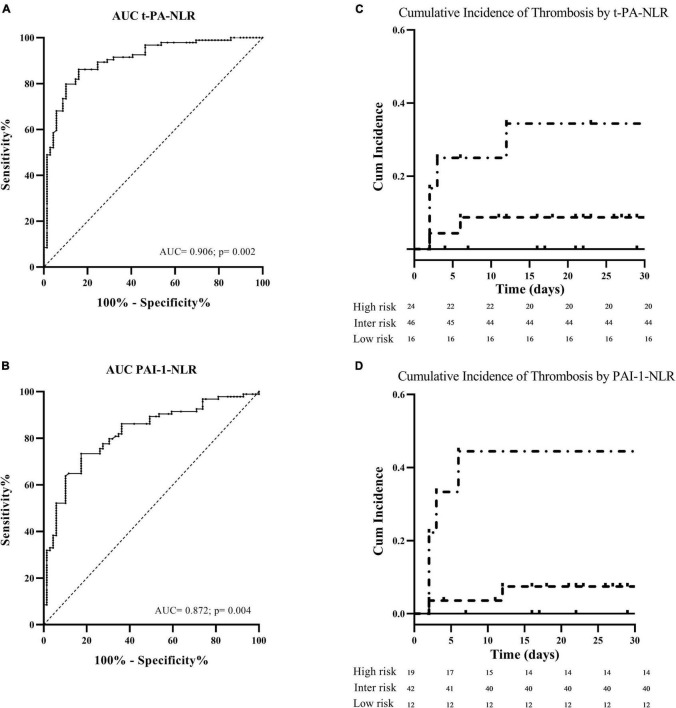
Tissue plasminogen activator-NLR and PAI 1-NLR as independent risk factors for thrombosis. On the left part of the figure, the receiver operating characteristic (ROC) curve analyses generated to determine the predictive accuracy for thrombosis of the combination of the neutrophil to lymphocyte ratio (NLR) with t-PA **(A)** or PAI-1 **(B)** values measured at enrollment. On the right part of the figure, the thrombotic risk stratification by the Kaplan–Meier survival curves in relation to score values of NLR plus t-PA **(C)** and NLR plus PAI-1 **(D)** are shown.

A multivariate Cox regression analysis was then performed starting from a model that included the three significant independent variables predictive for thrombosis (i.e., PAI-1, t-PA, and NLR) and clinical covariates (i.e., age, gender, COVID-19 severity, concomitant anticoagulation, PAFI). After backward selection, we identified the combination of PAI-1 together with NLR (HR: 1.056, IC 95%: 1.056–1.093; *p* = 0.002) (AUC = 0.872; *p* = 0.004), or t-PA together with NLR (HR: 1.125, IC 95%: 1.125–1.209; *p* = 0.002) (AUC = 0.906; *p* = 0.002), as independent risk factors for thrombosis (Figures 3A,B). Based on these results, two risk scores were created by assigning increasing points to the independent variables according to the selected cut-off values, i.e., 1 point for NLR >5, 1 point for t-PA or PAI-1 >25th percentile and 2 points for t-PA or PAI-1 >75th percentile, with the sum establishing the risk category. Higher the sum, higher the risk. Kaplan–Meier curves according to these groups are shown in Figures 3C,D. The cumulative incidence of thrombosis by t-PA plus NLR score was 0%, 9% (95% CI: 3–20%), and 33% (95% CI: 7–62%) for the for the low, intermediate, and high-risk group, respectively, providing an HR of 5.1 (CI: 95%: 1.104–24.2; *p* = 0.037). Regarding the PAI-1 plus NLR score, the cumulative incidence of thrombosis by was 0%, 7% (95% CI: 2–17%), and 44% (95% CI: 12–77%) for the for the low, intermediate, and high-risk group, respectively (HR: 8.3, CI: 95%: 1.6–43; *p* = 0.011).

### Predictors of Hemorrhagic Events During Follow-Up

During follow-up, after a median time of 12 days (range: 2–28 days), bleeding was recorded in 14 patients (Table 2). All the events were major according to the ISTH classification, and included six gastrointestinal, five central nervous system, and three urinary tract bleedings. At the time of the event, 12 patients were on anticoagulant treatment, that was LMWH at therapeutic dose in 6, LMWH at prophylactic dose in 4, UFH at therapeutic dose in 1, and DOAC in 1 patient.

Differently from the patients that developed a thrombotic event, measurement of anti-FXa levels at enrollment showed values within the expected ranges, as follows: 0.32 IU/mL (95% IC: 0.15–0.49) in patients on prophylactic LMWH, 0.42 IU/mL (95% IC: 0.13–1.1) on therapeutic LMWH, and 0.35 IU/mL in the 1 patient on therapeutic UFH. Neither age, gender, platelet count, hemoglobin concentration, comorbidities, or severity of the disease were associated with bleeding events by multivariable regression analysis.

Of note, among the different hemostatic variables analyzed, significantly lower levels of FXIII were detected in subjects who experienced major bleeding as compared to those who did not bleed (41%, CI: 95%: 18–74 vs. 63%, CI: 95%: 23–99; *p* = 0.001), and, by Cox regression univariable analysis, we found that higher FXIII levels were significantly associated with lower rate of bleeding events during follow-up (HR: 0.963; CI: 0.937–0.990; *p* = 0.008). According to the median value of FXIII distribution in the group of patients (i.e., 59%), a significantly lower cumulative incidence of bleeding was observed in subjects with levels ≥60% as compared to patients with levels ≤59% (8 vs. 60%; HR: 0.078; CI: 0.010–0.587; *p* = 0.013) (Figure 2D).

## Discussion

In this study we performed an extensive characterization of the hemostatic alterations by both global and specific assays in a cohort of patients hospitalized for COVID-19 associated pneumonia at two hospitals of the Bergamo area (Italy). This study was performed to clarify mechanisms underlying the coagulopathy in patients with different severity of the disease and to identify biomarkers potentially predictive of thrombotic and hemorrhagic complications to be utilized in large clinical studies.

Our study cohort consisted of 101 patients, with a median age of 67 years (range: 51–87 years). In this cohort we assessed the occurrence of thrombosis and bleeding during hospitalization. Specifically, among the enrolled patients, we registered a total of 53 events: 36 (7 during follow-up) were of thrombotic origin and 17 (14 during follow-up) were bleeding events.

At study enrollment 86% of the patients were receiving some anticoagulant strategy at prophylactic or therapeutic doses, that was heparin in most cases. Measurement of heparinemia showed levels of anti-FXa below the expected target in about 47% of subjects, including the seven patients that developed a thromboembolic event during follow-up. The occurrence of thromboembolic events in COVID-19 patients despite heparin thromboprophylaxis (“heparin resistance”) has been widely discussed by previous studies (1, 27–29). Some authors detected this heparin resistance *in vitro*, as non-inhibition of thrombin generation potential despite heparin therapy in hospitalized COVID-19 patients (25, 40).

The evaluation of the global hemostatic profile by ROTEM showed the presence of an elevated *in vitro* prothrombotic potential in the overall cohort of patients compared to controls, as assessed by thromboelastometry. This assay represents the dynamic analysis of specific aspects of hemostasis, and particularly, in our study, compared to controls, patients displayed shorter CFT and higher MCF values. These signs of hypercoagulability by ROTEM testing are not novel, and our results are consistent with the findings of studies showing shorter CFT and higher MCF in COVID-19 patients (25, 41–43).

To identify the main alterations in coagulation factors levels possibly responsible for the increased global procoagulant potential, we measured the activity of several coagulation factors. Our results showed significantly increased levels of FV, FVIII, FIX, FXI, and fibrinogen, all possibly contributing to the enhanced hemostatic potential detected by thromboelastometry. Indeed, statistical analyses revealed significant correlations between higher FVIII and fibrinogen levels with shorter CFT and higher MCF values. Of note, among all the coagulation factors, only FXIII was significantly reduced in patients, mainly in the ICU group. FXIII circulates in plasma as an inactive precursor and is activated by thrombin. After activation the enzyme stabilizes fibrin clots by cross-linking fibrin monomers, which increase the mechanical strength of the clot, increases resistance to fibrinolysis by cross-linking antifibrinolytic proteins to fibrin, including α2-antiplasmin, and increase platelet adhesion to the wounded tissues (44, 45). Decrease in FXIII in our patients may suggest a consumption of this factor, secondary to blood clotting activation and fibrin formation. However, we cannot exclude that the reduction in FXIII may be also due to a proteolytic digestion by thrombin, as well as by proteolytic enzymes released by activated granulocytes (46, 47).

The *in vitro* increased hemostatic potential by thromboelastometry in our patients was associated with the occurrence of an *in vivo* hypercoagulable state, as shown by the detection of increased F1 + 2 and D-dimer plasma levels, with the highest values of D-dimer observed in ICU subjects. While D-dimer has been largely investigated in this condition (13, 41, 48), and correlated with mortality from COVID-19 (25, 37, 49, 50), less information is available about F1 + 2, a biomarker of *in vivo* thrombin generation (51). Interestingly, levels F1 + 2 were strongly associated with levels of IL-6, a pleotropic cytokine released by several cell types, including dendritic cells and macrophages of the innate immune system, that plays an important role in the activation and regulation of the immune response (52). In addition, IL-6 has been implicated in the progression of several viral infections, including SARS-CoV-2. Inflammatory parameters’ analysis demonstrated significantly increased CRP, procalcitonin and NLR levels, especially in the ICU patients.

Of note, both CRP and procalcitonin inversely correlated with PC activity, while CRP and NLR were directly correlated with fibrinogenemia, providing additional evidence of the influence of inflammatory status on coagulation pathways also in this setting.

Regarding the concentrations of fibrinolytic proteins, t-PA, and its inhibitor PAI-1, were both elevated in our cohort compared to normal controls, without significant differences between ICU and non-ICU subjects. Both t-PA and PAI-1 are proteins of endothelial origin which are released upon proinflammatory stimuli. In our patients, the plasma levels of these two proteins well correlated to each other (*r* = 0.573), as reported in another cohort of hospitalized COVID-19 patients (53). PAI-1 is generally present in molar excess to t-PA and controls the amount of free-active t-PA, both in plasma and on endothelial cells surface, by establishing a t-PA-PAI-1 complex. Consequently, high plasma concentrations of PAI-1 are directly associated to reduced fibrinolysis and increased risk for thrombosis (54). In previous studies in patients with ARDS, high PAI-1 levels has been associated with the severity of the respiratory injury and poorer prognosis (26, 27, 55). This finding was also proved in patients with severe COVID-19, in whom elevated levels of both t-PA and PAI-1 were associated with worse respiratory status, while high levels of t-PA only were strongly associated with death (53). Interestingly, in our study, we could identify high baseline PAI-1 and t-PA levels as independent predictors of thrombosis during follow-up. This association further supports the central role of endothelial dysfunction and PAI-1 at the intersection of pulmonary and cardiovascular complications in COVID-19 infection (40, 42), suggesting the targeted inhibition of PAI-1 as a novel therapeutic option to improve outcomes beyond thrombosis in these patients. Together with these two fibrinolytic biomarkers, we found that also a high NLR value at enrollment was independently associated with thrombosis at follow-up. NLR is found increase in severe inflammation, stress, injury, trauma or major surgery, or cancer, and is a prognostic marker of morbidity or mortality in several pathological conditions (39), including COVID-19 (56–58). Interestingly, we found that the combination of pathological values of NLR (i.e., NLR >5) with increased levels of t-PA or PAI-1 was able to better identify patients at different risk of thrombosis, as compared to the stratification capacity of each independent predictors.

Major bleeding during follow-up occurred in 14 patients, all but 2 on anticoagulant drugs that consisted of therapeutic doses of LMWH in 75% of cases. Bleeding patients were characterized by significantly lower FXIII levels at enrollment compared to patients who did not bleed. Furthermore, by Kaplan–Meier analysis, we could identify low FXIII level (i.e., ≤59%, the median value of the cohort) as an independent risk factor for bleeding during follow-up. Low FXIII activity levels were recently reported in a small group of COVID-19 patients hospitalized in ICU, with gradual decline during their hospitalization, related to a mechanism of consumption (59). In our study we confirm this finding but, more importantly, we could correlate for the first time the decrease in FXIII with an increased risk of clinically relevant bleeding. Non-immune-mediated causes of acquired FXIII deficiency are more common than deficiencies secondary to autoantibodies, however, they rarely lead to life-threatening bleeding, and are due to increased consumption or decreased synthesis with activity levels in general ranging from 20 to 70% (60). In our patients the combination of low FXIII levels and concomitant heparin administration at therapeutic dose might be responsible of the bleeding manifestation. Furthermore, it should be also considered that FXIII not only is involved in fibrin stabilization, but it is also able to modulate inflammation, endothelial permeability, reduce multiple organ dysfunction, and trauma-hemorrhagic shock (45, 61, 62). It is possible that all these additional activities can be as well impaired in the presence of low levels of this enzyme, further contributing to the onset of bleeding episodes.

Our study presents some limitations. First, we performed a measurement of the biomarkers only at study enrollment. A longitudinal evaluation might provide the temporal changes of biomarker levels and their possible relevance. In addition, due to the small sample size, the results on thrombotic and bleeding complication prediction should be considered hypothesis-generating. A strength of our study is that, beside thrombosis, we have also evaluated the bleeding events, and in literature data on hemorrhagic complications in COVID-19 during hospitalization are few. In addition, this is a comprehensive analysis of blood coagulation determinants collected in a prospective series of patients that reveals some interesting novel insights in COVID-19 associated coagulopathy, such as the low levels of FXIII, and its potential value as predictive biomarker for bleeding.

In conclusion, our study provides an overview of the hemostatic alterations underlining the hypercoagulable state in hospitalized COVID-19 patients, even on thromboprophylaxis treatment. These hemostatic abnormalities are exacerbated by the severity of the disease and strongly correlate with the proinflammatory status, demonstrating the link between coagulation and inflammation. Hypercoagulability was supported by an imbalance between increased, rather than consumption of procoagulant factors, i.e., FV, FVIII, FIX, FXI, and fibrinogen, and decreased or normal natural coagulation inhibitors, i.e., antithrombin, PC. Among biomarkers, increased PAI-1 and t-PA, together with a high NLR, were associated with thrombotic events, while decreased FXIII with bleeding during hospitalization. This explorative study poses the bases for the evaluation of these biomarkers in larger prospective cohorts.

## Data Availability Statement

The raw data supporting the conclusions of this article will be made available by the authors, without undue reservation.

## Ethics Statement

The studies involving human participants were reviewed and approved by the ethics committee of Papa Giovanni XXIII Hospital. The Ethics Committee waived the requirement of written informed consent for participation.

## Author Contributions

MM and AF designed and supervised the study. LR, SG, ES, CV, FS, and CA performed the research. FR, EB, MR, SF, AD’A, FS, and LL supervised the clinical study management. PG-R and MM performed the statistical analysis and contributed to the data interpretation. PG-R, MM, and AF wrote the manuscript. LR and GG contributed to the data interpretation and wrote the manuscript. All authors read the final version of the manuscript and approved it prior to submission.

## Conflict of Interest

The authors declare that the research was conducted in the absence of any commercial or financial relationships that could be construed as a potential conflict of interest.

## Publisher’s Note

All claims expressed in this article are solely those of the authors and do not necessarily represent those of their affiliated organizations, or those of the publisher, the editors and the reviewers. Any product that may be evaluated in this article, or claim that may be made by its manufacturer, is not guaranteed or endorsed by the publisher.
